# Patient-Identified Problems and Influences Associated With Diagnostic Delay of Acromegaly: A Nationwide Cross-Sectional Study

**DOI:** 10.3389/fendo.2021.704496

**Published:** 2021-10-21

**Authors:** Kailu Wang, Xiaopeng Guo, Siyue Yu, Lu Gao, Zihao Wang, Huijuan Zhu, Bing Xing, Shuyang Zhang, Dong Dong

**Affiliations:** ^1^ The Jockey Club School of Public Health and Primary Care, Faculty of Medicine, The Chinese University of Hong Kong, Hong Kong, Hong Kong, SAR China; ^2^ Department of Neurosurgery, Peking Union Medical College Hospital, Chinese Academy of Medical Sciences and Peking Union Medical College, Beijing, China; ^3^ Key Laboratory of Endocrinology of Ministry of Health, Peking Union Medical College Hospital, Chinese Academy of Medical Sciences and Peking Union Medical College, Beijing, China; ^4^ China Pituitary Disease Registry Center, Beijing, China; ^5^ China Pituitary Adenoma Specialist Council, Beijing, China; ^6^ Department of Endocrinology, Peking Union Medical College Hospital, Chinese Academy of Medical Sciences and Peking Union Medical College, Beijing, China; ^7^ China Alliance of Rare Diseases, Beijing, China; ^8^ Department of Cardiology, Peking Union Medical College Hospital, Chinese Academy of Medical Sciences and Peking Union Medical College, Beijing, China; ^9^ Shenzhen Research Institute, The Chinese University of Hong Kong, Shenzhen, China

**Keywords:** acromegaly, diagnostic delay, misdiagnosis, pre-diagnostic advice, patient experience, comorbidity

## Abstract

**Background:**

Insidious-onset acromegaly may easily be overlooked by non-specialists of acromegaly and cause diagnostic delay. This study aims to examine the association between diagnostic delay and advice from doctors before any confirmed diagnosis and subsequent comorbidities, and elicit patient-perceived reasons for misdiagnoses.

**Methods:**

An online nationwide cross-sectional study was conducted through China Acromegaly Patient Association. Growth Hormone (GH) and Insulin-like Growth Factor 1 (IGF-1) levels at diagnosis and cancerous, endocrine-metabolic, musculoskeletal, cardiovascular, respiratory, and psychiatric comorbidities were reported by patients. The association between diagnostic delay and pre-diagnostic advice from doctors as well as subsequent comorbidities after diagnosis were examined.

**Results:**

In total, 447 valid responses were collected. Overall, 58.8% patients experienced misdiagnoses, and 22.6% had diagnostic delay. Before arriving at any diagnosis, patients without treatment (adjusted odds ratio [AOR]: 3.66, 95% confidence interval [CI]: 1.30-10.33) or receiving treatment to symptoms only (AOR: 7.05, 95%CI: 4.09-12.17) had greater chance of being misdiagnosed, and hence had diagnostic delay. Patients believed insufficient specialists, limited awareness of acromegaly of non-specialists and poor doctor-patient communications were major reasons of misdiagnosis. Diagnostic delay were associated with higher GH level at diagnosis and endocrine-metabolic, musculoskeletal and cardiovascular comorbidities (all P<0.05).

**Conclusions:**

Suboptimal pre-diagnostic advice for patients, reflecting limited awareness of acromegaly among non-specialists, may delay the diagnosis and increase comorbidities. Feedbacks on the patients’ final diagnosis from specialists to non-specialists should be considered, and doctor-patient communication and clinical decision-making process should be improved. Comorbidities should be screened and monitored particularly for patients with diagnostic delay.

## Introduction

Acromegaly is a rare chronic condition caused by the excessive secretion of Growth Hormone (GH) and Insulin-like Growth Factor 1 (IGF-1) (1). Elevated levels of GH and IGF-1 lead to a series of symptoms and comorbidities including enlarged hands and feet; facial feature changes; cardiovascular, musculoskeletal, endocrine, and metabolic disorders; and, cancer (2–4). However, the insidious onset of these symptoms and the overlapping clinical manifestations with other common conditions increases the risk of the condition being overlooked in medical consultations, especially by doctors who are not specialists in this area (5, 6). This results in difficulties in diagnosing acromegaly which may eventually cause misdiagnosis and lengthy durations before diagnosis that last for approximately 4.5–5 years from symptom onset to the diagnosis of acromegaly (7, 8).

Diagnostic delay or long duration before diagnosis, suggesting a delay in initiation of management of their hormone level and detection of their comorbidities, might cause worse health outcomes of patients. A longitudinal study in Sweden found that a longer diagnostic delay (defined as the time between the first onset of comorbidity and the diagnosis of acromegaly) led to increased mortality and a greater number of comorbidities (9). Other than physical health, delayed diagnosis may also have a negative impact on the quality of life and psychosocial functions of daily life (10, 11). Therefore, early diagnosis is essential for the future health status of patients with acromegaly (12).

In recent studies, the reasons for the diagnostic delay during the process of arriving at the correct diagnosis of acromegaly have been explored. In a few qualitative studies, the advice provided, or decisions and responses made by doctors, especially non-specialists, before the diagnosis could be the reasons for the misdiagnosis and the long diagnostic durations (13, 14). Additionally, it reminds us that patient-reported experiences and perceptions could be essential in identifying problems in medical consultation. Therefore, this study aimed to determine and quantify the association between the pre-diagnostic advice of doctors and diagnostic durations and elicit patient perceptions of the reasons for misdiagnosis. This study also aimed to examine the association between diagnostic durations and comorbidities after diagnosis, as no study of this scale has been performed in China.

## Methods

A nationwide cross-sectional survey was conducted in patients with acromegaly in China between 17 December 2019 and 6 January 2020. This study was approved by ethics committees from the Chinese University of Hong Kong and Peking Union Medical College Hospital (Reference number: SBRE-18-268 and SK-814). Informed consent was obtained from each participant prior to commencement of the survey.

### Study Participants and Data Collection

The participants of the study were recruited through the China Acromegaly Patient Association (CAPA), a non-profit organization established by patients with acromegaly and their families in 2012 that has a nationwide network of patients. The survey targeted patients with acromegaly. For the study, only adult patients with acromegaly were included in the analysis. In the survey, a link for the web-based questionnaire was sent to each patient by the staff of CAPA through its patient network *via* instant messengers (e.g. QQ) and social media (e.g. WeChat). It was a self-administered survey in which a staff member from CAPA and a member of the research team were available online to answer the questions from the survey participants. The online survey platform was designed to guide participants to avoid missing questions unintentionally. An information sheet related to this study was provided at the beginning where the participants indicated their consent to participating the survey. All the data collected by this survey were retrieved from the online survey platform after completion, and the data were protected by passwords.

### Variables

This survey focused on seven major factors. They included: 1) diagnostic information: calendar year of the first medical consultation for the condition and calendar year of receiving the diagnosis of acromegaly and whether the condition were misdiagnosed as other diseases; 2) pre-diagnostic advice from a doctor when the patient did not receive a confirmed diagnosis from the doctor; 3) initial symptoms resulting from acromegaly; 4) GH and IGF-1 levels at diagnosis and comorbidities including cancer, endocrine-metabolic, musculoskeletal, cardiovascular, respiratory, and psychiatric conditions at the time of survey (**Supplementary File**); 5) whether they received surgery, medication and/or radiotherapy after diagnosis, and the GH level at the latest follow-up to indicate whether or not the condition was under control (1.0 ng/mL as cut-off) (15); 6) their perceptions of reasons for the misdiagnosis and their source of information of the hospital that gave them the diagnosis of acromegaly; and, 7) age and sex of the patient. For this study, the patients who received the diagnosis of acromegaly more than one year after their first medical consultation for this condition were defined as those who experienced ‘diagnostic delay’. The pre-diagnostic advice from doctors before any confirmed diagnosis was obtained (irrespective of whether the diagnosis was right or wrong) comprised three major types, namely ‘without any treatment or advice’, ‘receive treatments to symptoms only based on doctor’s personal experience’, and ‘being referred or recommended to other doctors or hospitals as the doctor cannot make a diagnosis’. The number of initial symptoms was revealed from the patient’s responses to a list of selected symptoms (**Supplementary File**).

This survey was part of the 2019 General Social Survey on People with Rare Diseases in China. The design, implementation, and quality control of the survey were monitored by a committee comprising clinical specialists on acromegaly, managers and staff of CAPA, and research team members with multidisciplinary backgrounds. The questionnaire was refined and finalized based on a three-round pilot survey of over 10 participants.

### Statistical Analysis

The demographic and clinical characteristics of the patients were compared between the patients who experienced and did not experience diagnostic delays and misdiagnosis using the chi-square test for categorical variables and independent t-test or Wilcoxon rank-sum test for continuous variables. Two multiple logistic regression models for misdiagnosis and diagnostic delay were used to examine their associations with pre-diagnostic advice separately, with adjustment for demographics and number of initial symptoms. Those with missing values for misdiagnosis and diagnostic duration were excluded from the analysis. As there were very few members of CAPA who were not diagnosed with acromegaly, this study only included patients who had been diagnosed with acromegaly, while the patients who had already visited doctors for this condition but had not been diagnosed with acromegaly were not approached for the survey. This may lead to a selection bias that underestimates the likelihood of diagnostic delay. In the study sample, the closer the calendar year of the first medical consultation was to the survey date, the less likely the patients experienced a diagnostic delay. Therefore, the calendar year of the first medical consultation was also included as a covariate in both the regression models to adjust for this potential bias and for the improvement in diagnostic technology throughout the year.

Multiple linear regression was used to examine the association of diagnostic delay with GH and IGF-1 levels (in logarithm form) at diagnosis, and multiple logistic regression was used for cancerous, endocrine-metabolic, musculoskeletal, cardiovascular, respiratory, and psychiatric conditions at the time of the survey. The GH and IGF-1 levels and comorbidities were dependent variables which stood for adverse outcomes of acromegaly whereas diagnostic delay, type of treatment received, GH level at the latest follow-up, smoking, and demographics were independent variables. Moreover, patients’ perceptions on reason for misdiagnosis and their sources of information on the hospital that gave them the diagnosis of acromegaly were summarized. Kaplan–Meier (KM) curves were also plotted with log rank test to summarize the length of diagnostic delay in the subgroups with different pre-diagnostic advice and adverse outcomes, which was defined as the number of years between the first medical consultation and the diagnosis of acromegaly with a half-year adjustment. SPSS 26.0 (IBM Corp, NY USA) and Stata 15.0 (StataCorp, College Station, TX USA) was used for statistical analysis.

## Results

### Demographic and Clinical Characteristics

A total of 474 patients completed the questionnaire. Among them, 454 adults were diagnosed with acromegaly while others who were also diagnosed with pituitary gigantism were excluded. Among the 454 patients with acromegaly, 7 patients provided invalid responses regarding the year of the first medical consultation and the year of diagnosis (e.g., the year of diagnosis was earlier than their first consultation). Therefore, 447 patients were included in the analysis (**Table 1**). Of them, 59.3% were female, 76.8% were below 40 years of age at diagnosis, and 72.0% were from 31-50 years old at the time of the survey. More than half of them (n=263, 58.8%) experienced misdiagnoses before receiving a diagnosis of acromegaly and the remaining 41.2% of the study sample (n=184) did not experience misdiagnosis. There were 22.6% of them (n=101) experienced diagnostic delay (i.e. over one year between first medical consultation and diagnosis of acromegaly) and 77.4% of them (n=346) without such diagnostic delay. The median diagnostic duration of them was 0.5 year and the mean (SD) was 1.4 (2.4) years. Further, there were 6.7% of them without treatment by the doctors, while 36.7% of them received treatment to symptoms only and 38.5% were referred or recommended to other doctors or hospitals. Mean (SD) of GH and IGF-1 level at diagnosis was 46.6 (102.4) ng/ml [median (IQR): 19.1 (35.0) ng/ml] and 694.7 (347.2) ng/ml [median (IQR): 676.5 (437.0) ng/ml], respectively. At time of the survey, patients reported to have endocrine-metabolic (61.1%), psychiatric (40.7%), musculoskeletal (35.1%), cardiovascular (32.0%) and respiratory conditions (18.1%), and cancer (7.2%).

**Table 1:** Demographical and clinical characteristics of participants with or without experience of misdiagnosis and diagnostic delay.

	Experience of misdiagnosis					Experience of diagnostic delay							Total	
	No		Yes			No		Yes						
	N/mean	%/SD	N/mean	%/SD	P value^1^	N/mean	%/SD	N/mean		%/SD		P value^1^	N/mean	%/SD
**Age at the time of survey ^2^**	40.2 yrs	9.7 yrs	38.4 yrs	9.1 yrs	0.050	39.1 yrs	9.4 yrs	39.5 yrs		9.3 yrs		0.723	39.2 yrs	9.4 yrs
Below 30 yrs	18	9.8	39	14.8	0.298	46	13.3	11		10.9		0.914	57	12.8
31-40 yrs	81	44.0	121	46.0		154	44.5	48		47.5			202	45.2
41-50 yrs	53	28.8	67	25.5		93	26.9	27		26.7			120	26.8
51+ yrs	32	17.4	36	13.7		53	15.3	15		14.9			68	15.2
**Age at diagnosis ^2^**	36.1 yrs	9.8 yrs	33.9 yrs	9.0 yrs	0.016*	34.8 yrs	9.5 yrs	34.8 yrs		9.0 yrs		0.977	34.8 yrs	9.4 yrs
Below 30 yrs	53	28.8	115	43.7	0.014*	131	37.9	37		36.6		0.379	168	37.6
31-40 yrs	80	43.5	95	36.1		132	38.2	43		42.6			175	39.2
41-50 yrs	37	20.1	37	14.1		62	17.9	12		11.9			74	16.6
51+ yrs	14	7.6	16	6.1		21	6.1	9		8.9			30	6.7
**Sex**														
Male	94	51.1	88	33.5	<0.001**	147	42.5	35		34.7		0.159	182	40.7
Female	90	48.9	175	66.5		199	57.5	66		65.4			265	59.3
**No. of initial symptoms^2^**	6.9	3.2	9.3	3.8	<0.001**	8.1	3.6	9.0		4.1		0.033*	8.3	3.7
**Years between first doctor visit and the survey^3^**	5.0 yrs	6.0 yrs	6.0 yrs	4.0 yrs	0.009*	4.5 yrs	5.0 yrs	8.0 yrs		6.0 yrs		<0.001**	5.0 yrs	5.0 yrs
**Misdiagnosis**														
No	–	–	–	–	–	161	46.5	23		22.8		<0.001**	184	41.2
Yes	–	–	–	–		185	53.5	78		77.2			263	58.8
**Pre-diagnostic advice: without prescribing any treatment or advice**														
No	178	96.7	239	90.9	0.015*	330	95.4	87		86.1		0.001*	417	93.3
Yes	6	3.3	24	9.1		16	4.6	14		13.9			30	6.7
**Pre-diagnostic advice: treatments to symptoms only**														
No	155	84.2	128	48.7	<0.001**	236	68.2	47		46.5		<0.001**	283	63.3
Yes	29	15.8	135	51.3		110	31.8	54		53.5			164	36.7
**Pre-diagnostic advice: referring or recommending to other doctors/hospitals**														
No	105	57.1	170	64.6	0.105	203	58.7	72		71.3		0.022*	275	61.5
Yes	79	42.9	93	35.4		143	41.3	29		28.7			172	38.5
**Surgery**														
No	16	10.7	11	4.6	0.020*	18	6.0	9		9.7		0.224	27	6.9
Yes	134	89.3	231	95.5		281	94.0	84		90.3			365	93.1
(missing)	34	–	21	–		47	–	8		–			55	–
**Medications**														
No	48	32.0	67	27.7	0.362	91	30.4	24		25.8		0.392	115	29.3
Yes	102	68.0	175	72.3		208	69.6	69		74.2			277	70.7
(missing)	34	–	21	–		47	–	8		–			55	–
**Radiotherapy**														
No	107	71.3	138	57.0	0.004*	183	61.2	62		66.7		0.342	245	62.5
Yes	43	28.7	104	43.0		116	38.8	31		33.3			147	37.5
(missing)	34	–	21	–		47	–	8		–			55	–
**GH level at diagnosis ^2^**	36.6 **ng/ml**	80.4 **ng/ml**	53.3 **ng/ml**	114.4 **ng/ml**	0.007*	43.4 **ng/ml**	105.1 **ng/ml**	57.5 **ng/ml**		91.7 **ng/ml**		0.003*	46.6 **ng/ml**	102.4 **ng/ml**
**IGF-1 level at diagnosis ^2^**	695.7 **ng/ml**	378.1 **ng/ml**	694.0 **ng/ml**	327.3 **ng/ml**	0.793	686.1 **ng/ml**	352.4 **ng/ml**	724.7 **ng/ml**		328.8 **ng/ml**		0.218	694.7 **ng/ml**	347.2 **ng/ml**
**GH level at the latest follow-up ^2^**	18.50 **ng/ml**	166.1 **ng/ml**	9.1 **ng/ml**	61.1 **ng/ml**	0.442	14.6 **ng/ml**	129.4 **ng/ml**	6.7 **ng/ml**		23.4 **ng/ml**		0.622	12.8 **ng/ml**	114.6 **ng/ml**
**< 1.0 ng/mL**	53	28.8	80	30.4	0.713	104	30.1	29		28.7		0.795	133	29.8
**≥ 1.0 ng/mL**	131	71.2	183	69.6		242	69.9	72		71.3			314	70.3
**Musculoskeletal conditions**														
No	134	72.8	156	59.3	0.003*	236	68.2	54		53.5		0.006*	290	64.9
Yes	50	27.2	107	40.7		110	31.8	47		46.5			157	35.1
**Cardiovascular conditions**														
No	143	77.7	161	61.2	<0.001**	241	69.7	63		62.4		0.168	304	68.0
Yes	41	22.3	102	38.8		105	30.4	38		37.6			143	32.0
**Type 2 Diabetes**														
No	161	87.5	225	85.5	0.555	295	85.3	91		90.1		0.213	386	86.4
Yes	23	12.5	38	14.5		51	14.7	10		9.9			61	13.6
**Other endocrine-metabolic conditions**														
No	112	60.9	137	52.1	0.066	200	57.8	49		48.5		0.098	249	55.7
Yes	72	39.1	126	47.9		146	42.2	52		51.5			198	44.3
**Respiratory conditions**														
No	158	85.9	208	79.1	0.067	282	81.5	84		83.2		0.702	366	81.9
Yes	26	14.1	55	20.9		64	18.5	17		16.8			81	18.1
**Cancer**														
No	172	93.5	243	92.4	0.662	318	91.9	97		96.0		0.156	415	92.8
Yes	12	6.5	20	7.6		28	8.1	4		4.0			32	7.2
**Psychiatric conditions**														
No	116	63.0	149	56.7	0.176	206	59.5	59		58.4		0.840	265	59.3
Yes	68	37.0	114	43.4		140	40.5	42		41.6			182	40.7
**Total**	184	100.0	263	100.0		346	100.0	101		100.0			447	100.0

*P < 0.05, **P < 0.001.

^1^P values was calculated using Chi-square test, independent t-test and Wilcoxon rank-sum test.

^2^The values under column ‘N’ are mean, and the values under column ‘%’ are standard deviation.

The values under column “N” were median, and the values under column “%” were interquartile range (IQR).

### Association of Misdiagnosis and Diagnostic Delay With Pre-Diagnostic Advice

**Table 1** shows the univariate analysis of the characteristics of the patients who experienced (n=263) or did not experience (n = 184) misdiagnosis, as well as in relation to those who had (n = 101) and did not have (n = 346) diagnostic delay. It is observed that those with experience of misdiagnosis were significantly more likely than those without misdiagnosis to be younger at diagnosis (Mean ± SD: 33.9 ± 9.0 *vs*. 36.1 ± 9.8 years, P=0.016), women (P<0.001), have more initial symptoms (P<0.001), have closer/later calendar year of first medical consultation (P=0.009), receive no treatment (P=0.015) or treatment to symptoms only (P<0.001) before diagnosis of acromegaly, have surgery (P=0.020) or radiotherapy (P=0.004) for treatment, have higher GH level at diagnosis (P=0.007), have musculoskeletal (P=0.003) and cardiovascular conditions (P<0.001) at the time of survey.

The univariate analysis also revealed that those who experienced diagnostic delay were more likely to receive no treatment or advice (13.9% *vs* 4.6% among those who did not have diagnostic delay, P=0.001) or receive treatment to symptoms only (53.5% *vs* 31.8%, P<0.001) before receiving any diagnosis, while they were less likely to be referred or recommended to other hospitals or doctors (28.7% *vs* 41.3%, P=0.022). The patients with experience of diagnostic delay were also more likely to have more initial symptoms (P=0.033), have closer/later calendar year of first medical consultation (P<0.001), experience misdiagnosis (P<0.001), have higher GH level at diagnosis (P=0.003), and have musculoskeletal conditions at the time of survey (P=0.006). However, there was no significant difference of age at diagnosis between patients with and without diagnostic delay (Mean ± SD: 34.8 ± 9.0 *vs*. 34.7 ± 9.5 years, P=0.977).

On adjusting for covariates (**Table 2**), misdiagnosis was significantly associated with the pre-diagnostic advice without prescribing any treatment or advices [adjusted odds ratio (AOR): 3.66, 95% confidence interval (CI): 1.30-10.33] and pre-diagnostic advice of treatment to symptoms only (AOR: 7.05, 95%CI: 4.09-12.17), as well as age between 31-40 years compared to age before 300 years and female sex. On the other hand, diagnostic delay was significantly associated with an earlier calendar year of first doctor visit (AOR: 0.83, 95%CI: 0.78-0.88), misdiagnosis (AOR: 2.18, 95%CI: 1.17-4.07), and treatment to symptoms only (AOR: 1.93, 95%CI: 1.07-3.50). In addition, even though the association was not statistically significant, those without treatment were slightly more likely to had a diagnostic delay (AOR: 2.30, 95%CI: 0.92-5.78, P=0.076).

**Table 2:** Association of experience of misdiagnosis and diagnostic delay with pre-diagnostic advice.

	Misdiagnosis		Diagnostic delay	
	Adjusted OR^1^	95%CI^2^	Adjusted OR	95%CI
**Age at diagnosis**				
Below 30	(Reference)		(Reference)	
31-40	0.57*	(0.34, 0.94)	1.45	(0.82, 2.55)
41-50	0.61	(0.32, 1.16)	1.16	(0.52, 2.57)
51+	0.60	(0.24, 1.49)	2.07	(0.70, 6.08)
**Sex**				
Male	(Reference)		(Reference)	
Female	1.85*	(1.18, 2.90)	1.29	(0.76, 2.20)
**No. of initial symptoms**	1.19	(1.12, 1.27)	1.02	(0.95, 1.09)
**Calendar year of first doctor visit**	0.98	(0.93, 1.03)	0.83*	(0.78, 0.88)
**Misdiagnosis**				
No	–		(Reference)	
Yes	–		2.18*	(1.17, 4.07)
**Pre-diagnostic advice: without prescribing any treatment or advice**				
No	(Reference)		(Reference)	
Yes	3.66*	(1.30, 10.33)	2.30	(0.92, 5.78)
**Pre-diagnostic advice: treatments to symptoms only**				
No	(Reference)		(Reference)	
Yes	7.05*	(4.09, 12.17)	1.93*	(1.07, 3.50)
**Pre-diagnostic advice: referring or recommending to other doctors/hospitals**				
No	(Reference)		(Reference)	
Yes	1.48	(0.91, 2.43)	0.82	(0.45, 1.50)

*P < 0.05.

^1^OR, odds ratio;

^2^CI, confidence interval.

**Table 3** shows the length of diagnostic delay by different pre-diagnostic advices and comorbidities. The patients without receiving any treatment or advice from doctors (P<0.001) or those who received treatment to symptoms only (P=0.019) were found to have longer diagnostic delay (**Table 3** and **Supplementary Figure A1–A3**). Regarding comorbidities, the diagnostic delay was significantly associated with musculoskeletal conditions (P = 0.010) and cardiovascular conditions (P = 0.017) (**Table 3** and **Supplementary Figure A4–A10**).

**Table 3:** Length of diagnostic delay by different pre-diagnostic advices and comorbidities.

	Time to diagnosis^1^ by pre-diagnostic advice (years)				
	No/No such advice		Yes/received such advice		log rank test P value^2^
	Mean	SD	Mean	SD
**Without prescribing treatment or advice**	1.2	2.0	3.3	5.4	<0.001**
**Treatments to symptoms only**	1.2	2.5	1.6	2.2	0.019*
**Referring or recommending to other doctors/hospitals**	1.5	2.6	1.1	2.1	0.080
	**Time to diagnosis by comorbidities at time of this survey (years)**				
	**Without this condition**		**With this condition**		**log rank test P value**
	**Mean**	**SD**	**Mean**	**SD**	
**Musculoskeletal conditions**	1.1	2.0	1.7	3.1	0.010*
**Cardiovascular conditions**	1.2	1.9	1.8	3.3	0.017*
**Type 2 Diabetes**	1.4	2.5	1.0	1.9	0.220
**Other endocrine-metabolic conditions**	1.2	2.0	1.6	2.9	0.073
**Respiratory conditions**	1.4	2.6	1.1	1.7	0.306
**Cancer**	1.4	2.4	1.2	2.4	0.526
**Psychiatric conditions**	1.3	2.5	1.4	2.3	0.625
**Total**	1.4	2.4			

*P < 0.05, **P < 0.001.

^1^Time to diagnosis was defined as time between first medical consultation and diagnosis of acromegaly; ^2^P values came from log-rank test of the time to diagnosis by different subgroups. Relevant Kaplan-Meier curves can be found in Appendix.

### Patient’s Perception on Reasons of Misdiagnosis

From the patients’ perspective (**Table 4**), issues that can be ascribed to doctors and doctor-patient communication, including ‘insufficient doctors with this specialty’, ‘low awareness and limited knowledge of this disease’, ‘poor doctor-patient communication’ and ‘insufficient time of doctors for details of patients’ condition and background’, were the most important reasons for misdiagnosis according to 72.5% of all the participants. Issues that can be ascribed to patients, including ‘low frequency of medical consultation’, ‘lack of information source for hospital/doctors with specialties’, ‘reluctance to share information with doctors’, ‘lack of knowledge of one’s own conditions’ and ‘afraid of prejudice and discrimination to patients with rare diseases’, were reported to be the most important reasons by 13.9% participants, and the second most important reason according to 40.1% participants. Compared to these two major reasons, fewer participants believed the nature of medical science and current diagnostic technology (8.3%), issues relevant to hospitals (3.8%) or financial reasons (1.6%) lead to misdiagnosis.

**Table 4:** Patient perceived importance of reasons of misdiagnosis and information sources of hospitals giving the correct diagnosis.

Reasons	Rank of importance of reasons (n=447)					
	Most important reason		2^nd^ most important reason		3^rd^ most important reason	
	N	%	N	%	N	%
Issues ascribed to doctors and their communication with patients^1^	324	72.5	74	16.6	61	13.6
Issues ascribed to patients^2^	62	13.9	179	40.1	92	20.5
Issues ascribed to medical science and diagnostic test^3^	37	8.3	129	28.8	176	39.4
Issues ascribed to hospitals^4^	17	3.8	43	9.6	84	18.8
Financial issues^5^	7	1.6	22	4.9	32	7.2
Sources of information	Rank of importance of information source of the hospitals giving the correct diagnosis (n=447)					
	Primary source		2nd source		3rd source	
	N^6^	%	N	%	N	%
Selecting it based on the hospital’s reputation in general	193	43.2	21	4.7	3	0.7
Recommended by family members/friends	94	21.0	18	4.0	6	1.3
Recommended by doctors	53	11.9	18	4.0	4	0.9
Internet search	41	9.2	32	7.2	7	1.6
Patient group	24	5.4	9	2.0	6	1.3

^1^Issues of doctors and their communication with patients included ‘insufficient doctors in this specialty’, ‘low awareness of this disease’, ‘limited knowledge of this disease which led to misjudgment’, ‘poor communication between patients and doctors’ and ‘doctors do not have enough time to ask details of the patients’ condition and background’.

^2^Issues of patients included ‘low frequency of medical consultation’, ‘lack of valid information source to know about hospital/doctors specialized in this kind of disease’, ‘reluctance to share some information with doctors’, ‘lack of knowledge to one’s own conditions’ and ‘prejudice and discrimination against patients with rare diseases’.

^3^Issues of medical science and diagnostic tests included ‘substantial knowledge gaps between different specialties’, ‘limited communications among specialties’, ‘limited methods/equipment for diagnosis’ and ‘sensitivity and accuracy of diagnostic test not ideal’.

^4^Issues of hospital included ‘no screening tool provided by the hospitals for this disease’ and ‘difficulties in getting reservation for an expert’.

^5^Financial issues included ‘high charge of diagnostic test’ and ‘need to travel around other cities to get diagnosed’.

^6^The numbers of patients in the column of primary source of information for the hospital do not add up to 447 as the responses of 42 patients were missing values.

### Patent’s Information Source on Hospitals Giving the Correct Diagnosis

The patients selected the hospitals that gave them the correct diagnosis of acromegaly primarily based on their general reputations (43.2%), followed by recommendations by their family members or friends (21.0%) and doctors (11.9%) (**Table 4**). Internet search (9.2%) and patient groups (5.4%) played less significant roles in helping patients to find such hospitals.

### Association of Diagnostic Delay With GH and IGF-1 Level and Comorbidities

Elevated GH and IGF-1 level and comorbidities were considered as adverse outcomes of diagnostic delay. Patients who experienced diagnostic delay had a higher GH level at diagnosis (57.5 *vs* 43.4 ng/ml among those without diagnostic delay, P=0.003), but their IGF-1 levels were similar (unadjusted mean: 724.7 *vs* 686.1 ng/ml among those without diagnostic delay, P=0.218; age- & sex-adjusted IGF-1 standard deviation score [SDS] ± SD): 4.52 ± 2.71 *vs* 4.19 ± 2.89 among those without diagnostic delay, P=0.395) (16). Those with diagnostic delay were also more likely to have musculoskeletal conditions at time of this survey (46.5% *vs* 31.8% among those without diagnostic delay, P=0.006) (**Table 1**).

From the multiple regressions for GH and IGF-1 level (**Table 5**), GH level at diagnosis of those with diagnostic delay was around 43% higher than those without the delay (Coefficient: 0.43, 95%CI: 0.13-0.74, P=0.005) after adjusting for age, sex and smoking, but the delay was not significantly associated with the IGF-1 SDS (Coefficient: 0.38, 95%CI: -0.37-1.14, P=0.319) with adjustment of the covariates. As for comorbidities at the time of survey, patients with diagnostic delay were more likely to have musculoskeletal conditions (AOR: 1.78, 95%CI: 1.06-2.97, P=0.026) and endocrine-metabolic conditions other than diabetes (AOR: 1.67, 95%CI: 1.03-2.70, P=0.038) after adjusting for age, sex, smoking, GH level at the latest follow-up, and types of treatment received after being diagnosed with acromegaly. Besides these two types of comorbidities, patients with diagnostic delay were also more likely to have cardiovascular conditions (AOR: 1.62, 95%CI: 0.97-2.70, P=0.067), but less likely to be diagnosed with cancer (AOR: 0.23, 95%CI: 0.05-1.01, P=0.051) at the time of the survey after adjusting for aforementioned covariates, despite the fact that these two associations showed a slight trend toward statistical significance. However, there was no significant associations between diagnostic delay and type 2 diabetes (AOR: 0.68, 95%CI: 0.32-1.42, P=0.303).

**Table 5:** Association of diagnostic delay with GH and IGF-1 level and comorbidities.

	GH level at diagnosis^1^	IGF-1 SDS at diagnosis^1^	Musculoskeletal conditions	Cardiovascular conditions	Type 2 Diabetes	Other endocrine-metabolic conditions	Respiratory conditions	Cancer	Psychiatric conditions
	Coefficient^2^	Coefficient^2^	Adjusted OR^2^	Adjusted OR^2^	Adjusted OR^2^	Adjusted OR^2^	Adjusted OR^2^	Adjusted OR^2^	Adjusted OR^2^
**Diagnostic delay (‘No’ as reference)**									
Yes	0.43*	0.38	1.78*	1.62	0.68	1.67*	0.84	0.23	0.92
(95% CI^3^)	(0.13, 0.74)	(-0.37, 1.14)	(1.06, 2.97)	(0.97, 2.70)	(0.32, 1.42)	(1.03, 2.70)	(0.45, 1.57)	(0.05, 1.01)	(0.56, 1.50)
**Age at diagnosis^4^/at time of survey (Below 30 as reference)**									
31-40	-0.21	0.45	2.30*	2.74*	0.70	1.36	2.12	1.99	1.95*
(95%CI)	(-0.50, 0.07)	(-0.27, 1.17)	(1.10, 4.78)	(1.24, 6.06)	(0.32, 1.55)	(0.74, 2.48)	(0.83, 5.44)	(0.52, 7.59)	(1.06, 3.61)
41-50	-0.36	1.46*	7.03*	6.72*	0.66	1.71	3.55*	3.23	1.80
(95%CI)	(-0.73, 0.01)	(0.54, 2.38)	(3.21, 15.36)	(2.94, 15.39)	(0.27, 1.61)	(0.88, 3.30)	(1.35, 9.32)	(0.83, 12.54)	(0.92, 3.52)
51+	-0.46	2.24*	8.59*	7.74*	1.71	1.53	3.31*	1.99	1.56
(95%CI)	(-0.99, 0.06)	(1.01, 3.47)	(3.44, 21.45)	(3.01, 19.95)	(0.65, 4.48)	(0.68, 3.43)	(1.09, 10.01)	(0.37, 10.76)	(0.69, 3.53)
**Sex (Male as reference)**									
Female	0.05	-0.43	1.48	1.27	1.08	1.73*	0.93	3.14	2.81*
(95%CI)	(-0.25, 0.34)	(-1.16, 0.30)	(0.84, 2.61)	(0.72, 2.22)	(0.52, 2.25)	(1.03, 2.88)	(0.49, 1.77)	(0.98, 10.10)	(1.63, 4.84)
**Smoking (‘No’ as reference)**									
Yes	0.09	-0.25	1.20	1.50	1.40	1.42	1.74	4.46*	2.57*
(95%CI)	(-0.25, 0.44)	(-1.10, 0.61)	(0.62, 2.32)	(0.78, 2.87)	(0.61, 3.22)	(0.78, 2.61)	(0.86, 3.53)	(1.36, 14.63)	(1.37, 4.83)
**Surgery (‘No’ as reference)**									
Yes	–	–	0.89	0.86	1.00	1.14	0.88	2.07	0.74
(95%CI)	–	–	(0.37, 2.14)	(0.37, 2.05)	(0.30, 3.29)	(0.49, 2.67)	(0.33, 2.29)	(0.25, 17.20)	(0.32, 1.73)
**Medication (‘No’ as reference)**									
Yes	–	–	1.19	0.97	0.91	1.14	1.06	1.18	1.47
(95%CI)	–	–	(0.70, 2.04)	(0.57, 1.63)	(0.46, 1.82)	(0.70, 1.86)	(0.58, 1.95)	(0.45, 3.13)	(0.89, 2.41)
**Radiotherapy (‘No’ as reference)**									
Yes	–	–	1.88*	1.38	1.58	2.04*	0.89	1.79	1.43
(95%CI)	–	–	(1.16, 3.05)	(0.85, 2.24)	(0.85, 2.92)	(1.30, 3.18)	(0.51, 1.57)	(0.78, 4.10)	(0.92, 2.24)
**GH level at the latest follow-up (‘<1ng/mL as reference’)**									
**≥**1 ng/mL	–	–	1.73*	1.39	1.21	0.79	1.17	1.45	1.08
(95%CI)	–	–	(1.02, 2.95)	(0.83, 2.35)	(0.60, 2.42)	(0.49, 1.28)	(0.64, 2.13)	(0.54, 3.89)	(0.67, 1.75)

*P < 0.05. ^1^The GH level were transformed into logarithm form due to skewed distribution, while IGF-1 SDS represents its standard deviation scores. ^2^The figures in the table under GH and IGF-1 level are coefficients of linear regressions, and the others are adjusted odds ratios of logistic regression. ^3^CI, confidence interval; ^4^only regressions of GH and IGF-1 level used age at diagnosis while others used age at time of the survey.

## Discussion

This study highlighted suboptimal advices from non-specialists in acromegaly were associated with diagnostic delay, and found out how this delay is associated with increasing comorbidities at follow-up. The findings were obtained from the data collected from the first nationwide survey in China for patients with acromegaly. The study showed that different advices given by doctors before reaching a confirmed diagnosis were associated with different likelihoods of misdiagnosis and diagnostic delay. The findings suggested that the absence of treatments or advices could contribute to misdiagnosis, and eventually lead to diagnostic delay. The pre-diagnostic treatments for the symptoms of patients based on doctor’s personal experience, which could be based on misjudgments of the reasons of the clinical manifestations or merely targeted to the comorbidities caused by acromegaly, were more common and therefore had greater negative impacts on efforts to reach a correct diagnosis. It would not only lead to greater chances of subsequent misdiagnosis, but it could also result in a lengthy duration before reaching to a definite diagnosis of acromegaly.

These suboptimal pre-diagnostic advices provided by doctors reflected a lack of awareness related to acromegaly across different specialties in the medical community (8, 17). According to the patients in this survey, the main reasons for misdiagnosis included insufficient specialists; limited awareness and knowledge of acromegaly in doctors, especially in those who are non-specialists; poor communications between doctors and patients; and a lack of information sources for hospitals or doctors with this specialty. Moreover, most of the patients reported that the reason for them to eventually find the hospital where they received the correct diagnosis was because the hospital had a good reputation in general rather than by a clear referral recommendation given by prior consultations with non-specialists. These findings were similar to those of a qualitative study in France (14), where the patients reported that some physicians admitted their ignorance to the conditions and could not find the causes of their sign and symptoms, showing a lower awareness of acromegaly among these physicians, whereas some of them failed to listen to or believe the complaints made by the patients about their symptoms, indicating a doctor-patient communication problem.

Physicians in the primary care setting or in other specialties who were the first contact point for the patients were found to play significant roles in initiating the evaluation that led to diagnosis of acromegaly in the end (18). In light of the issues stated above, education and information dissemination of the disease should be made in doctors in primary care or in other specialties, including in those specialized in cancer, musculoskeletal, cardiovascular and respiratory diseases, which are the common comorbidities of acromegaly (19), to better understand the clinical manifestations of acromegaly in order to enhance their awareness of this disease and consider it in their differential diagnosis.

Given the significant impact of pre-diagnostic advice from doctors, improving of their clinical decision-making process could also be helpful in reducing the diagnostic durations. Retrospectively speaking, based on this study, it would be advisable for doctors to invite consultations of other doctors or to refer the patients to the specialists that they believe could give insights to their conditions when they could not reach to a confirmed diagnosis. However, this requires ways to help non-specialists realize that limitations might exist in their current diagnosis and treatment methods, which could enable them to consider for asking consultations or making referrals to specialists (20). Therefore, specialists can consider to provide feedbacks on the diagnosis and management of relevant patients to their primary care physicians or the doctors who gave the first medical consultation, to help them better understand this disease and inform their future decisions. A previous study also suggested establishment of informal communication between general physicians and specialists could lead to more appropriate referrals (21).

Clinical decisions should be based on adequate information (22), and considering the limitations in doctor-patient communication brought up by the patients, guidelines for the clinical practice of non-specialists should be updated in favor of enhancing communications between patients and doctors and between doctors with different specialties. In both this study and another study in the US (13), patients with acromegaly were reluctant to share information about their condition to their healthcare providers. However, a high discrepancy rate was observed between patient- and physician-reported clinical manifestations of the condition (23). Therefore, patients should be encouraged to share information of all of the persistent symptoms that they encountered, instead of sharing different symptoms with different doctors based on their own judgement and preference (13). On the doctors’ side, those who lack specialty in this area should consider recording proper and comprehensive notes of the clinical manifestations reported by the patients or those discovered during clinical examinations for the reference of patients’ future consultations, even if the diagnosis could not be reached at that moment. In addition, the development of rapid clinical case-finding tools, either using traditional methods or artificial intelligence (17, 24–26), could help doctors identify potential acromegaly from their comorbidities or symptoms.

Furthermore, the findings implied that the substantial adverse outcomes of elevated GH levels at diagnosis and subsequent endocrine-metabolic (except diabetes), musculoskeletal and cardiovascular comorbidities were associated with diagnostic delay. The increased comorbidities may reduce quality of life of the patients (27) and increase their mortality (28, 29). This result is similar to the findings from the study using Swedish registry data (9) that patients with longer diagnostic delay tended to have more musculoskeletal, endocrine-metabolic and cardiovascular comorbidities in univariate analysis, while the pattern of respiratory disease prevalence across different length of diagnostic delay was unclear. However, it was found in our study that those with cancer was less likely to experience diagnostic delay. In contrast, those with longer diagnostic delays were more likely to have cancer in the Swedish study. Due to the cross-sectional nature of this survey, cancer could be appeared prior to the diagnosis of acromegaly, and those with cancer might seek medical consultation more frequently and receive comprehensive physical examinations that lead to the diagnosis of acromegaly. In other studies, cancer, especially thyroid cancer, was found to be associated with acromegaly (30–32). Its relationship with diagnostic duration can be explored in future studies.

The median time from diagnosis of acromegaly to the survey was only 4-5 years, which was lower than the average follow-up durations of other studies ranged from 7-13 years (9, 28, 29, 33). The average age at diagnosis in this study [Mean (SD): 34.8 (9.4) years] was also lower than these previous studies (range: 42-51.8 years). Compared these previous studies with this survey, more patients were suffered from diabetes [16%-30% *vs* 16.8% in this survey (including both type 1 and type 2 diabetes)] (28, 29, 33), respiratory diseases (including chronic lung disease and sleep apnea, 18%-29% *vs* 18.1% in this survey) (28, 33) and cancer (8%-11% *vs* 7.2%) (33). Prevalence of comorbidities in these previous studies were higher than that in this study as the patients were younger at diagnosis in this study. Apart from this reason, the comorbidities might increase in longer term of follow-up, and the adverse effects of diagnostic delay might also be different then. Nevertheless, what can be told from this study was that in the short-to-medium term after the diagnosis of acromegaly, patients’ endocrine-metabolic, musculoskeletal and cardiovascular functions and biomarkers should be screened at diagnosis and monitored at follow-up in order to provide adequate and timely treatment to them (34, 35), particularly to those with diagnostic delay. Additionally, their mental health should also be considered during the treatment period due to the high prevalence of psychiatric conditions (40.7%), including depression and anxiety (36, 37), despite no difference found between those with and without diagnostic delays.

A few limitations of this study should be noted. First, this study adopted a cross-sectional design, one must exercise caution while drawing conclusions about causal relationships. Second, recall bias may exist as it asked the participants to recall their experience retrospectively, particularly for their initial symptoms. Third, selection bias also exists as those who have visited the doctors for this condition but have not received a confirmed diagnosis were not selected for the study. However, the calendar year of the first medical consultation was used as a covariate to reduce its influence, as stated in the Methods section. Selection bias was also caused by relatively younger age of the study sample at diagnosis, which may have fewer comorbidities compared with other studies using registry data. Lastly, whether or not the acromegaly is under control can affect the presence of comorbidities, so we used GH level at the latest follow-up before the survey as an indicator for controlled/uncontrolled condition. However, the GH level at the latest follow-up might not be representative for presence of uncontrolled conditions during the entire follow-up period after diagnosis. This issue can be addressed in longitudinal studies that recorded the GH level at multiple time points during the follow-up period.

In summary, before arriving at any diagnosis for patients with acromegaly, absence of treatment of any kind and treatments to symptoms only, reflecting the limited awareness and knowledge of acromegaly of non-specialists who were the first contact point of the patients and insufficient information for clinical decision-making due to poor doctor-patient communication, were associated with subsequent diagnostic delays and misdiagnosis. Educating non-specialists about acromegaly, disseminating feedbacks on the patients from specialists to non-specialists, and improving doctor-patient communication and clinical decision-making process might reduce suboptimal pre-diagnostic advice. Diagnostic delay was also associated with worse health status at later stage, including higher GH levels at diagnosis and higher prevalence of endocrine-metabolic, musculoskeletal and cardiovascular comorbidities at follow-up. Screening of these conditions at diagnosis and regular monitoring efforts at follow-up should be performed to provide timely treatment and management particularly for patients with diagnostic delay.

## Data Availability Statement

The datasets used and/or analyzed during the current study are available from the corresponding author on reasonable request.

## Ethics Statement

The studies involving human participants were reviewed and approved by Survey and Behavioural Research Ethics, The Chinese University of Hong Kong (SBRE-18-268), and PUMCH Ethics Committee, Peking Union Medical College Hospital (SK-814). The patients/participants provided their written informed consent to participate in this study.

## Author Contributions

KW, XG, SY, BX, SZ, and DD designed the study. KW, SY, HZ, and DD performed the survey and collected responses. BX, HZ, SZ, and DD supervised the implementation and quality control of the survey. KW performed the statistical analysis. KW and XG prepared the manuscript. BX, SZ, and DD revised the manuscript. All authors contributed to the article and approved the submitted version.

## Funding

This work was supported by the National Key Research and Development Program of China (Grant No. 2016YFC0901500) and the Center for Rare Diseases Research, Chinese Academy of Medical Sciences, Beijing, China (Grant No. 2016ZX310174-4). The funders did not involve in study design and collection, analysis, and interpretation of the results.

## Conflict of Interest

The authors declare that the research was conducted in the absence of any commercial or financial relationships that could be construed as a potential conflict of interest.

## Publisher’s Note

All claims expressed in this article are solely those of the authors and do not necessarily represent those of their affiliated organizations, or those of the publisher, the editors and the reviewers. Any product that may be evaluated in this article, or claim that may be made by its manufacturer, is not guaranteed or endorsed by the publisher.
