# Supplementary material for: Patient-Identified Problems and Influences Associated With Diagnostic Delay of Acromegaly: A Nationwide Cross-Sectional Study
Source: Front Endocrinol (Lausanne). 2021 Oct 21;12:704496. doi: 10.3389/fendo.2021.704496 (PMC8566913; doi:10.3389/fendo.2021.704496)

Supplementary Material

1. **List of initial symptoms and signs counted for variable ‘number of initial symptoms’**
2. Facial feature change;
3. Enlarged hands and feet;
4. Gigantism;
5. Palpitations;
6. Atrial fibrillation;
7. Spacing in the teeth;
8. Edge-to-edge bite or crossbite;
9. Joint pain;
10. Snoring;
11. Hyperhidrosis;
12. Sleep apnoea;
13. Hyperlipidemia;
14. Headache;
15. Vision loss;
16. Visual field defect;
17. Low back pain;
18. Hairy;
19. Acne;
20. Menstrual disorder or amenorrhoea;
21. Lactation;
22. Low sexual desire; and,
23. Sexual disorders.
24. **List of comorbidities included in the study**
    1. **Musculoskeletal conditions:** a) vertebral fracture, b) arthritis, c) carpal tunnel syndrome, and d) osteoporosis.
    2. **Cardiovascular conditions:** a) hypertension, b) coronary heart disease, c) cardiomyopathy, d) heart failure, e) valvular heart disease, f) arrhythmia, g) heart block, h) arteriosclerosis, and i) stroke.
    3. **Diabetes:** a) type 2 diabetes.
    4. **Other endocrine-metabolic conditions:** a) high cholesterol, b) hyperlipidaemia, c) thyroid disease, d) hyperuricemia, and e) multiple endocrine neoplasia type 1 (MEN-1).
    5. **Respiratory conditions:** a) chronic obstructive pulmonary disease (COPD), b) asthma, and c) sleep apnoea syndrome or nocturnal hypoxemia.
    6. **Cancer:** a) thyroid cancer, b) colonic cancer, c) breast cancer, and d) other cancers specified by the patients.
    7. **Psychiatric condition:** a) depression, b) anxiety, c) anorexia/bulimia, and d) other psychiatric conditions specified by the patients.
25. **Kaplan-Meier curves according to different pre-diagnostic advices**

Different length of diagnostic duration was found between patients with different pre-diagnostic advices (Supplementary figure A1-A3). Patients without treatment from doctors before any confirmed diagnosis was made (mean±SD: 3.3±5.4 years; median: 0.5 years, 25th percentile [Q1] - 75th percentile [Q3]: 0.5-4.5 years) had longer diagnostic duration than those who were not (mean±SD: 1.2±2.0 years; median: 0.5 years, Q1-Q3: 0.5 years; P<0.001). The patients who received treatment to symptoms only before any confirmed diagnosis was made (mean±SD: 1.6±2.2 years; median: 0.5 years, Q1-Q3: 0.5-1.5 years) also experience a longer diagnostic duration than others (mean±SD: 1.2±2.5 years; median: 0.5 years, Q1-Q3: 0.5 years; P=0.019). No significant difference in the diagnostic duration found between referral to other hospitals or doctors (mean±SD: 1.1±2.1 years; median: 0.5 years, Q1-Q3: 0.5 years) and no referrals (mean±SD: 1.5±2.6 years; median: 0.5 years, Q1-Q3: 0.5-1.5 years; P=0.080).

Supplementary figure A1. Whether with or without any treatment or advice before any confirmed diagnosis (log rank test P <0.001)


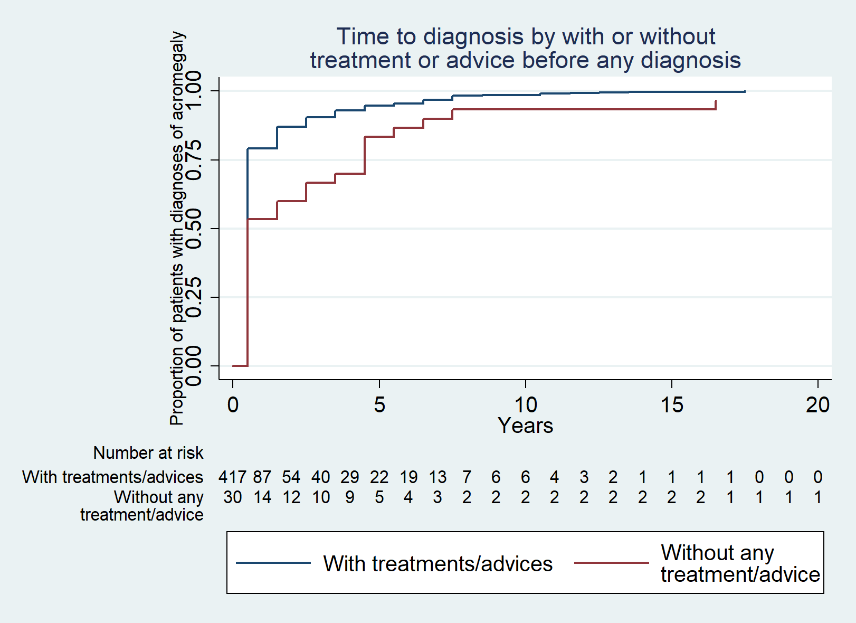


Supplementary figure A2. Whether received treatments to symptoms only before any confirmed diagnosis (log rank test P=0.019)


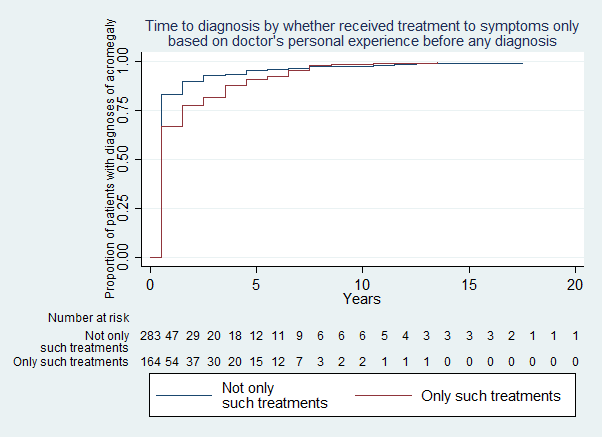


Supplementary figure A3. Whether being referred to other hospitals/doctors before any confirmed diagnosis (log rank test P = 0.080)


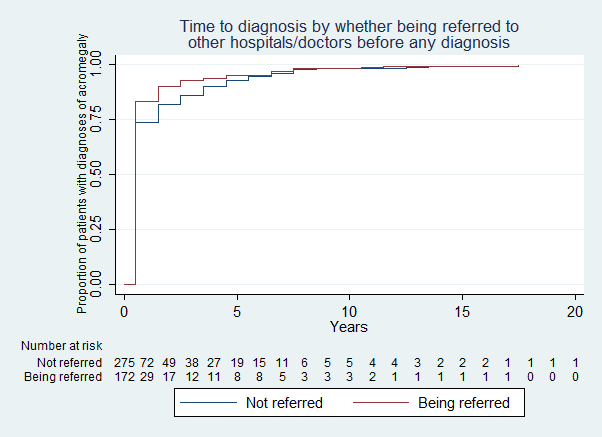


**4. Kaplan-Meier curves according to comorbidities at time of survey**

For length of diagnostic delay (Supplementary figure A4-A10, Appendix), it was found that patients with musculoskeletal conditions (mean±SD: 1.7±3.0 years; median: 0.5 years, Q1-Q3: 0.5-1.5 years) were more likely to have a longer diagnostic duration than those without these conditions (mean±SD: 1.1±2.0 years; median: 0.5 years, Q1-Q3: 0.5 years; P=0.010). A similar pattern was also observed in cardiovascular conditions (mean±SD: 1.8±3.3 years; median: 0.5 years, Q1-Q3: 0.5-1.5 years among those with these conditions vs. mean±SD: 1.2±1.9 years; median: 0.5 years, Q1-Q3: 0.5 years among those without these conditions; P=0.017).

Supplementary figure A4. Time to diagnosis by musculoskeletal conditions at the survey (long rank test P=0.010)


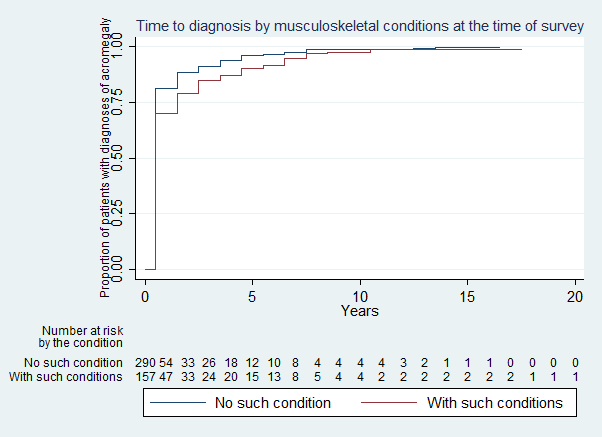


Supplementary figure A5. Time to diagnosis by cardiovascular conditions at the survey (long rank test P=0.017)


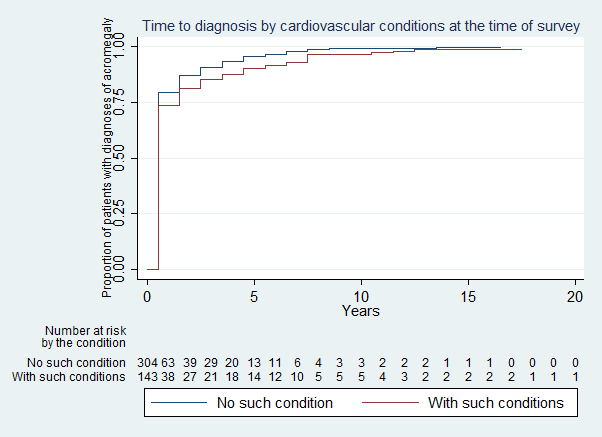


Supplementary figure A6. Time to diagnosis by type 2 diabetes at the survey (long rank test P=0.220)


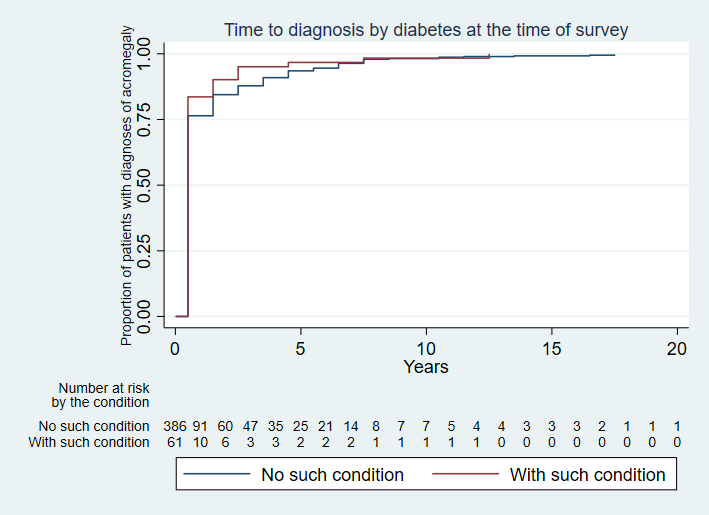


Supplementary figure A7. Time to diagnosis by endocrine-metabolic conditions (other than diabetes) at the survey (long rank test P=0.073)


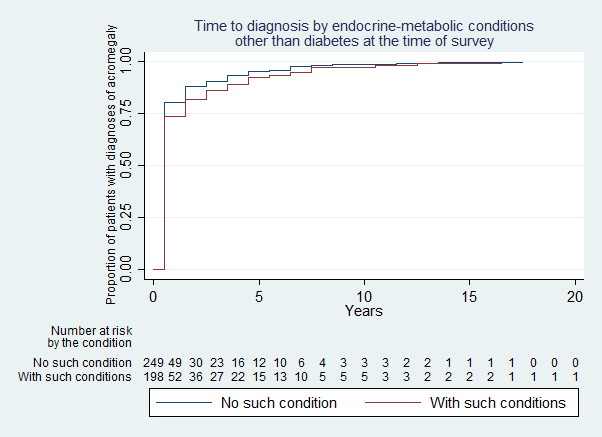


Supplementary figure A8. Time to diagnosis by respiratory conditions at the survey (long rank test P=0.306)


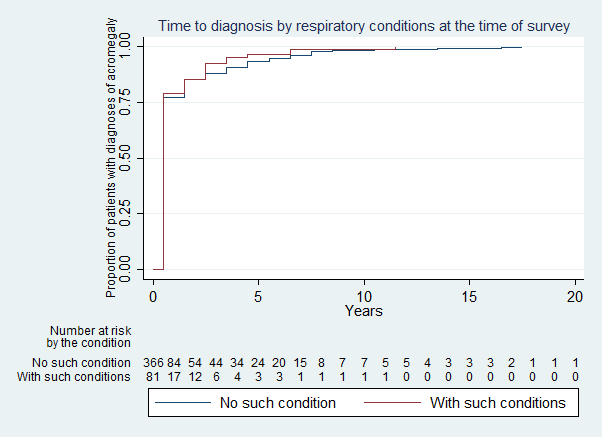


Supplementary figure A9. Time to diagnosis by cancer at the survey (long rank test P=0.526)


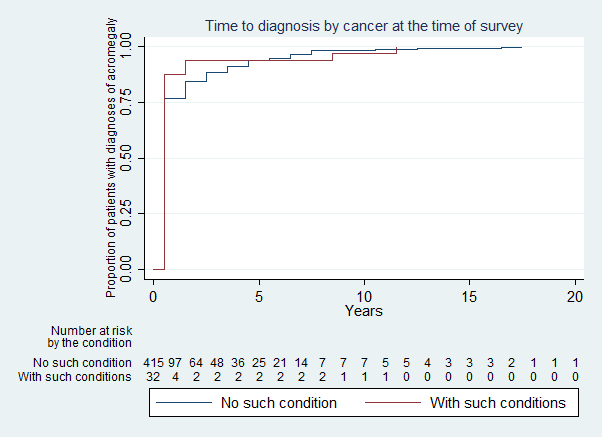


Supplementary figure A10. Time to diagnosis by psychiatric conditions at the survey (long rank test P=0.625)


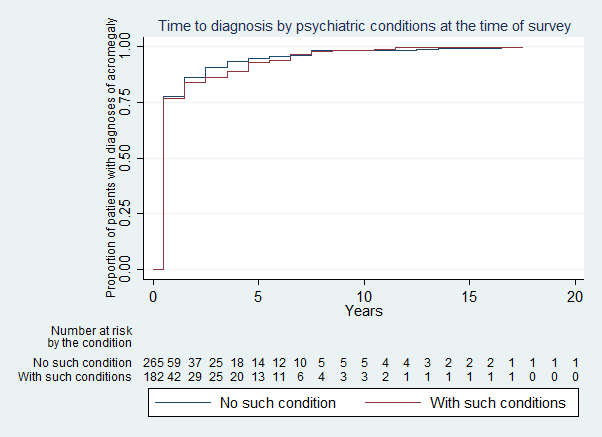

Supplement: Supplementary file 1 [file DataSheet_1.docx]
